# Interleukin-37 is involved in the immunopathogenesis of infectious mononucleosis

**DOI:** 10.1186/s13052-023-01498-5

**Published:** 2023-07-28

**Authors:** Mingsheng Zhao, Li Ma, Huihui Jiang, Yufeng Gu, Xin Yang, Riming Liu, Chengming Sun, Yulan Li

**Affiliations:** 1 Institute of Immunology and Molecular Medicine, Jining Medical University, Jining, China; 2 Center for Laboratory Diagnosis, Yantai Yuhuangding Hospital Affiliated to Qingdao University, Yantai, China

**Keywords:** Infectious mononucleosis, Interleukin-37, Epstein–Barr virus, CD3 + T cells

## Abstract

**Background:**

Multiple immunopathological responses to viruses are observed in infectious mononucleosis (IM), a manifestation of primary infection with Epstein-Barr virus (EBV). Protective effects of the negative immunoregulatory molecule interleukin-37 (IL-37) have been observed in various bacterial and viral infections. However, the function of IL-37 in IM remains unknown.

**Methods:**

Flow cytometry and enzyme-linked immunosorbent assay (ELISA) were used to determine the expression of IL-37 in the peripheral blood of patients diagnosed with IM, and the variation of lymphocyte subsets. Furthermore, the associations between IL-37 expression and the percentage of lymphocyte subgroups were analyzed.

**Results:**

Patients with IM had severe immune dysfunction. The control group had a lower expression of IL-37 than the patients with IM. There were significant associations between IL-37 expression and both the proportion of CD3^+^T cells and the ratio of CD3^+^CD4^+^ to CD3^+^CD8^+^T cells. Patients with higher levels of IL-37 expression had lower levels of the liver inflammation indicators, alanine aminotransferase (ALT) and aspartate aminotransferase (AST).

**Conclusions:**

IL-37 may affect the immune pathogenesis of patients with IM infected with EBV, and may have immunotherapeutic benefit for EBV-associated illnesses.

## Background

More than 90% of the adult population worldwide is infected by Epstein-Barr virus (EBV), however only a fraction develops infectious mononucleosis (IM). EBV infection is usually asymptomatic in the childhood [1, 2], while about 50% of older patients develop acute IM. EBV mainly targets human B-lymphocytes and nasopharyngeal epithelial cells. EBV infection with IM is invariably fatal and almost always has serious repercussions in immunocompromised hosts, caused by the immunopathological reactions of the virus (such as autoimmune hemolysis, airway obstruction from enlarged tonsils, splenic rupture, encephalitis, severe hepatitis, and myocarditis) [3].

There is no FDA or EMA-approved therapy for IM. Although certain antiviral drugs can reduce the EB viral load, they have no effect on clinical disease [4, 5]. Antiviral medications are ineffective in treating IM because the disease symptoms and signs are not caused by virus replication but by the immune system’s reaction to EBV-infected B cells that are circulating in the body. Therefore, the combined application of antiviral drugs and immunomodulatory drugs (such as corticosteroids and IFN-γ) may be effective. However, the underlying mechanisms by which the human immune system control IM are not well known.

Interleukin-37 (IL-37) was discovered in 2000, and was found to be a negative immunoregulatory molecule that belongs to the IL-1 family [6]. By modulating numerous inflammatory signaling pathways, IL-37 has a protective function in autoimmune and inflammatory illnesses, such as rheumatoid arthritis [7], colitis [8], psoriasis [9], obesity [10] and lipopolysaccharide (LPS)-induced endotoxemia [11]. Moreover, it inhibits tumor progression through immune regulation or directly suppresses the malignant behavior of tumor cells [12, 13]. However, whether IL-37 can treat IM remains unclear.

## Methods

### Subjects

This study recruited 61 patients with IM aged 1–15 years and 24 healthy individuals (HIs) from Yantai Yuhuangding Hospital associated with Qingdao University. The IM diagnostic criteria were previously described [14]. Patients with the classic triad of pharyngitis, fever and lymphadenopathy as presenting signs, were included.

Many potential infections with viruses or bacteria, such as herpes simplex virus, rubella virus, mycoplasma and chlamydia were ruled out by serological testing. In addition, children with persistent immunological and infectious illnesses were excluded. For the initial diagnosis, our medical center obtained peripheral blood samples in EDTA-K2 tubes. The peripheral blood sample was collected during the early stage of the disease and without any treatment.

### Flow cytometry

A Ficoll-Hypaque density gradient was utilized for the isolation of peripheral blood mononuclear cells (PBMCs) (Dakewei, China). The antibodies used for flow cytometry were as follows: CD19-APC, CD3-FITC, CD4-PE-Cy7, CD8-APC-Cy7 and CD16/CD56-PE (644,611, BD Biosciences). CD19^+^B cells, CD3^+^T cells, CD3^+^CD4^+^T cells, CD3^+^CD8^+^T cells, CD3^−^CD16^+^CD56 + NK cells, and CD3^+^CD16^+^CD56^+^NKT cells were defined through antibodies that were applied for staining cells. We employed isotype-matched Ab controls in every procedure pursuant to the manufacturer’s guidelines for each staining. We subsequently detected and analyzed the labeled cells using a FACS Canto II flow cytometer and Diva software (Becton Dickinson, Sparks, MD), respectively.

### EBV DNA quantification

As per the guidelines provided by the manufacturer, we performed an amplification of a 191 bp region of the *EBNA-1* gene using a commercial real-time PCR kit (BioQuant EBV, Biodiversity). This allowed us to calculate the amount of EBV-DNA in whole blood. For detecting the fluorescence signal, we used the ABI PRISM 7500 Sequencer Detection System (Applied Biosystems, USA).

### Wright staining

A blood smear was made on a glass slide. Slides were then stained using a standard protocol. Briefly, the blood smear was soaked with 300 µL Wright stain solution for 3 min. Thereafter, equal volume of PBS was added and gently shaken for 5 min. The liquid was removed and blotted. The stained lymphocytes were observed under a light microscope.

### ELISA

For in-depth analysis, we collected the serum samples and froze them at -80℃. As per the manufacturer’s guidelines, serum IL-37 levels were measured using IL-37 ELISA kits (R&D Systems, Minneapolis, USA).

### Statistical analysis

We used GraphPad Prism 8 for the statistical analysis (La Jolla, CA, USA), and Student’s *t-test* was used to determine the differences between the two groups. Spearman’s correlation was used to analyze each variable. The data are presented as mean ± standard deviation (SD). A *p* value < 0.05 was considered to be statistically significant.

## Results

### Immune dysfunction occurred in patients with IM

**Fig. 1:**
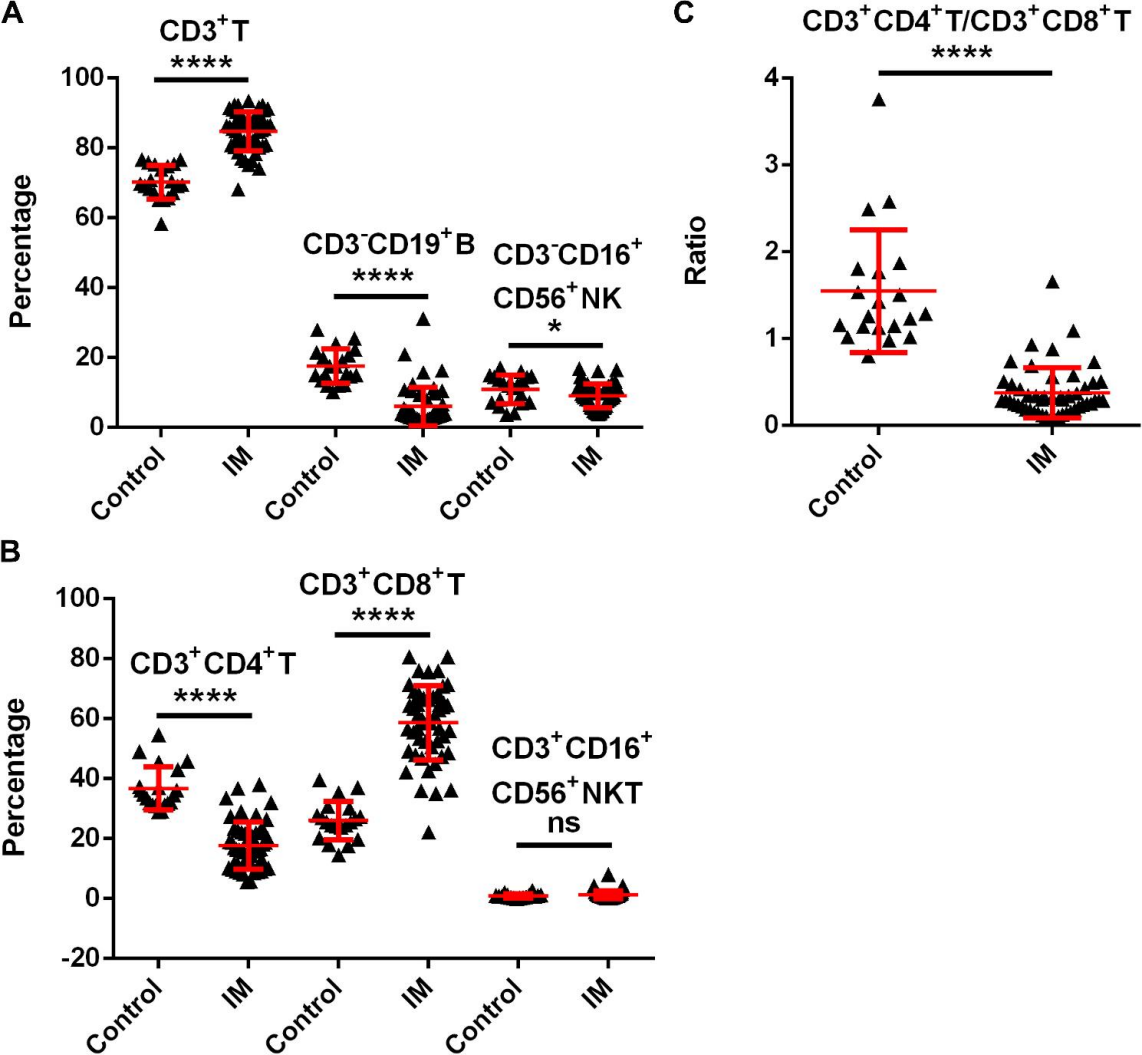
Immune disorders in patients with IM. The percentages of CD3^+^T, CD3^−^CD19^+^B, CD3^−^CD16^+^CD56^+^NK (**A**), CD3^+^CD4^+^T, CD3^+^CD8^+^T, CD3^+^CD16^+^CD56^+^NKT cells (**B**) among the controls (n = 24) and new onset patients with IM (n = 61) detected by flow cytometry. (**C**) The ratio of CD3^+^CD4^+^T to CD3^+^CD8^+^T cells in the controls and patients with IM. Data are expressed as mean ± SD, * p < 0.05, **** p < 0.0001, ns denotes *p* > 0.05

According to reports, EBV mainly attacks B-cells and permanently lodges itself as a dormant infection in resting memory B-cells. After the virus has replicated, numerous antigens are produced on the surface of the cell, which triggers a powerful cytotoxic response mediated primarily by T cells [3]. Compared to healthy controls, the frequency of CD3^+^T cells was significantly much higher in patients with IM while the frequency of CD3-CD19^+^B cells was significantly lower, which is in line with previous studies. In addition, the frequency of CD3^−^CD16^+^CD56^+^NK cells showed a slight decrease (Fig. 1A). More importantly, further experiments on the group of CD3^+^T cells revealed that the frequency of CD3^+^CD4^+^T (helper T cells, Th) and CD3^+^CD8^+^T (cytotoxic T cells, CTL) cells was higher in the opposite direction (Fig. 1B), which resulted in a significantly lower ratio of CD3^+^CD4^+^/CD3^+^CD8^+^T cells (Fig. 1C). These findings revealed that the patients’ immune function was damaged, which in turn implied a decrease in antiviral response and a worsening of the condition.

### IL-37 expression was upregulated in patients with IM

**Fig. 2:**
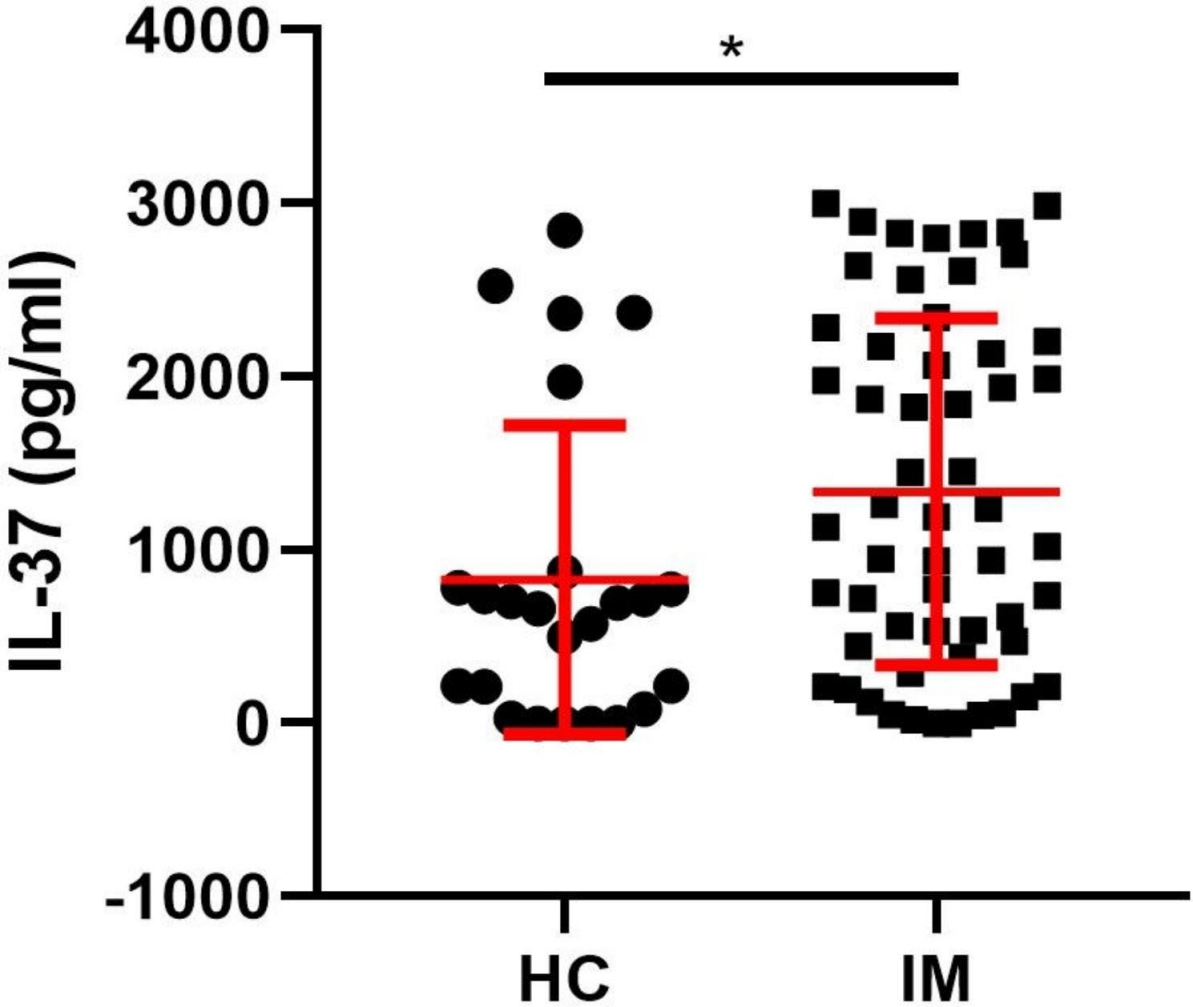
Elevated expression of IL-37 in patients with IM. IL-37 expression in the controls and patients with IM examined by ELISA. Data are expressed as mean ± SD, * p < 0.05

We utilized an enzyme-linked immunosorbent assay (ELISA) to evaluate IL-37 expression in the blood of patients with IM to determine how IL-37 affects IM. Comparison of IL-37 expression between patients with IM and healthy controls (Fig. 2) showed that patients with IM expressed higher IL-37 than controls.

### Expression of IL-37 was not associated with the viral load of EBV

**Fig. 3:**
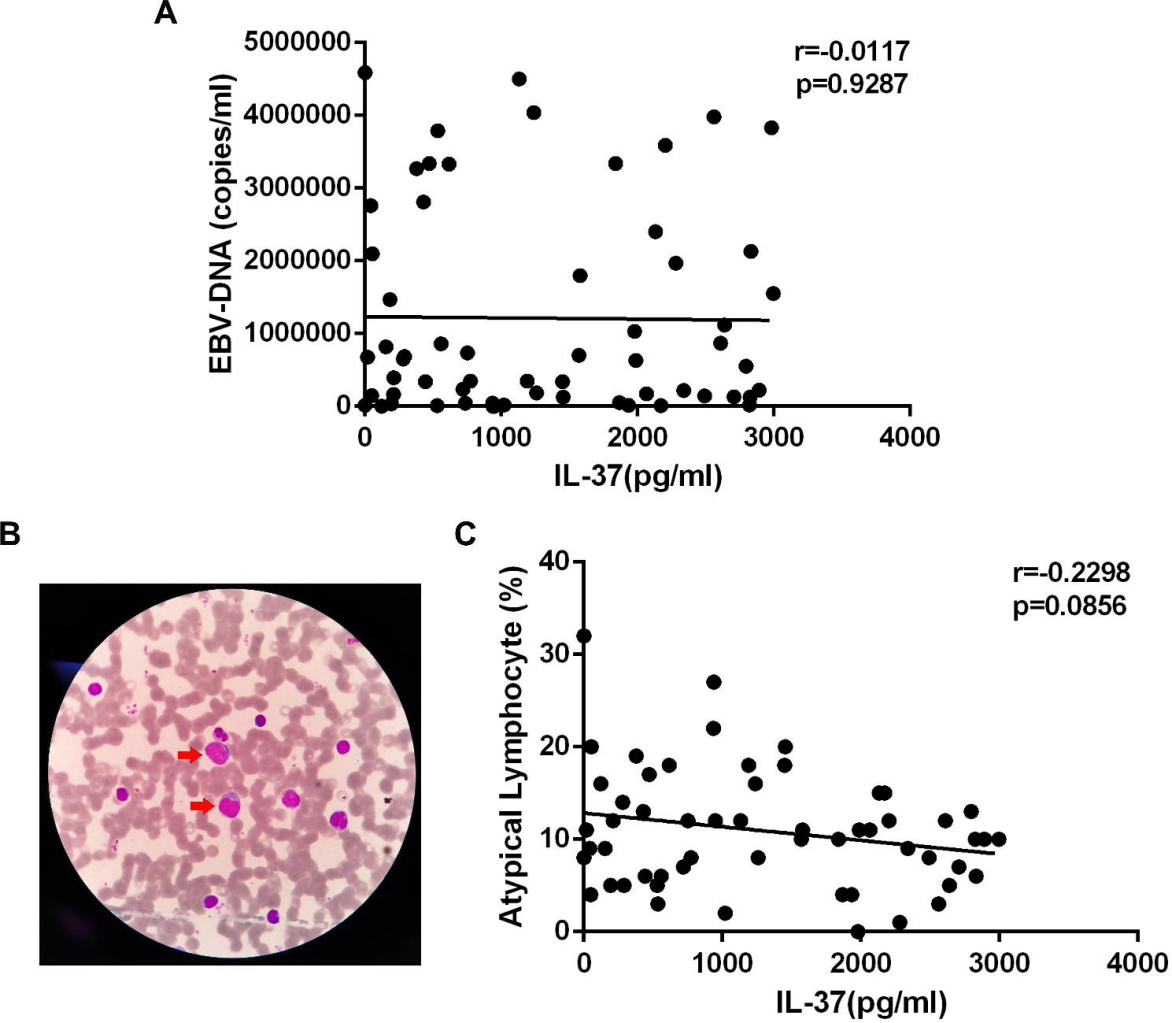
No significant correlation was observed between EBV-DNA copy number, the percentage of atypical lymphocytes and IL-37 expression in patients with IM. The correlation between EBV-DNA copy number (**A**), the percentage of atypical lymphocytes (**C**) and IL-37 expression. (**B**) Representative hematoxylin-eosin (H&E)–stained atypical lymphocyte in peripheral blood of patients with IM.

Viral load in the blood increases due to high levels of EBV replication in IM. Unexpectedly, our data showed no association between IL-37 expression and copies of EBV-DNA (Fig. 3A). Studies have indicated that IM is characterized by a robust T cell response that is mostly composed of activated CD8 + cytotoxic T cells (atypical lymphocytes) specific for latent viral antigens produced on EBV-infected B cells (Fig. 3B). Our subsequent investigation revealed a negative correlation between IL-37 expression and the number of atypical cells in peripheral blood, although without statistical significance (Fig. 3C).

### IL-37 expression was strongly related to lymphocyte frequency

**Fig. 4:**
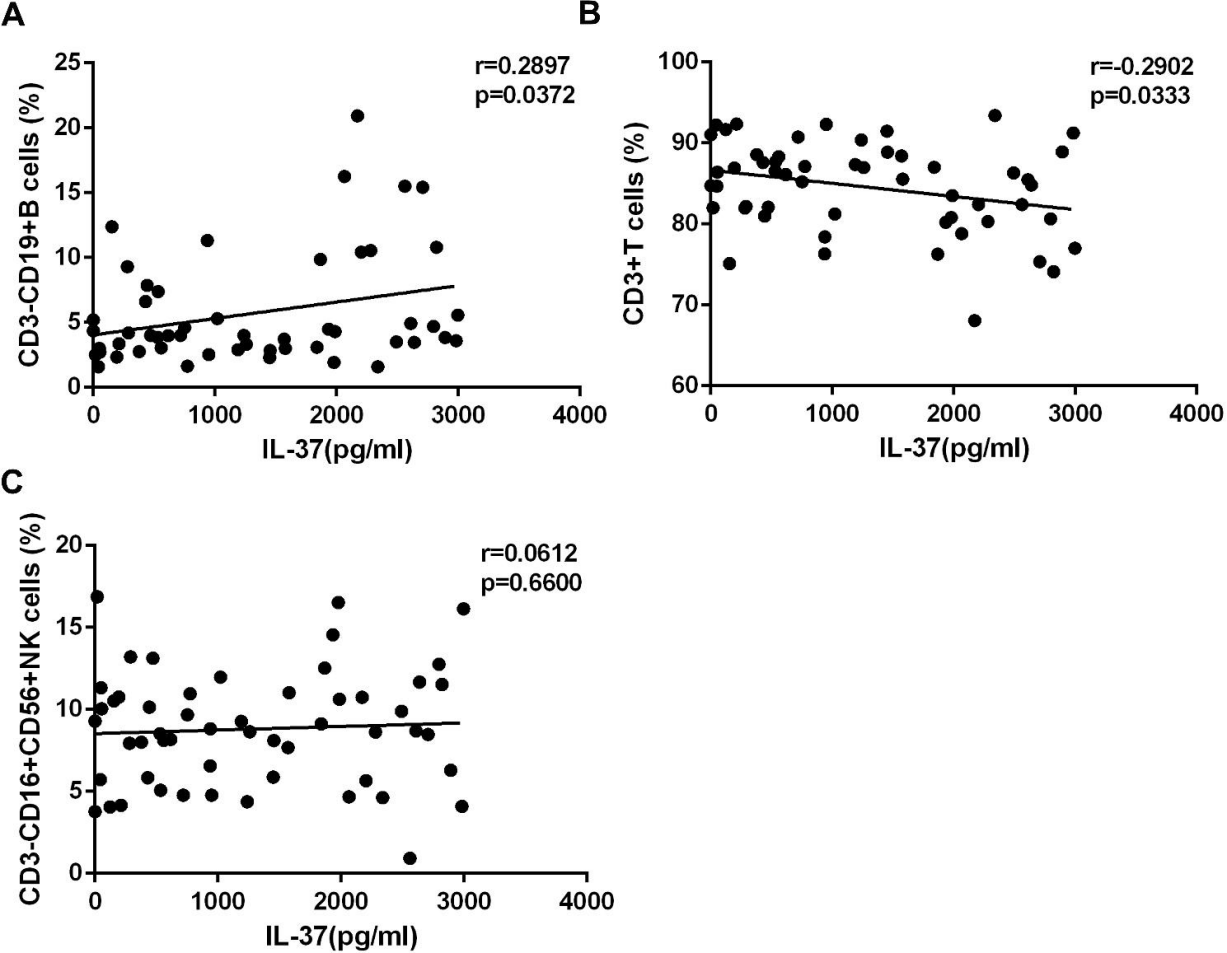
IL-37 expression was negatively correlated with CD3^+^ T cells. The correlation between the percentages of CD3^−^CD19^+^B (**A**), CD3^+^T (**B**), CD3^−^CD16^+^CD56^+^NK (**C**) and IL-37 expression

IL-37 is a natural negative immune regulator [6]. Increased IL-37 expression suggests that it may play a potential regulatory role in the immune dysfunction of patients with IM. The expression of IL-37 had a strong positive correlation with the frequency of CD3^−^CD19^+^B cells (Fig. 4A), but a significant negative correlation with the frequency of CD3^+^T cells (Fig. 4B). However, the expression of IL-37 was unrelated to CD3^−^CD16^+^CD56^+^NK cells (Fig. 4C).

### IL-37 might participate in the regulation of immune dysfunction in patients with IM

**Fig. 5:**
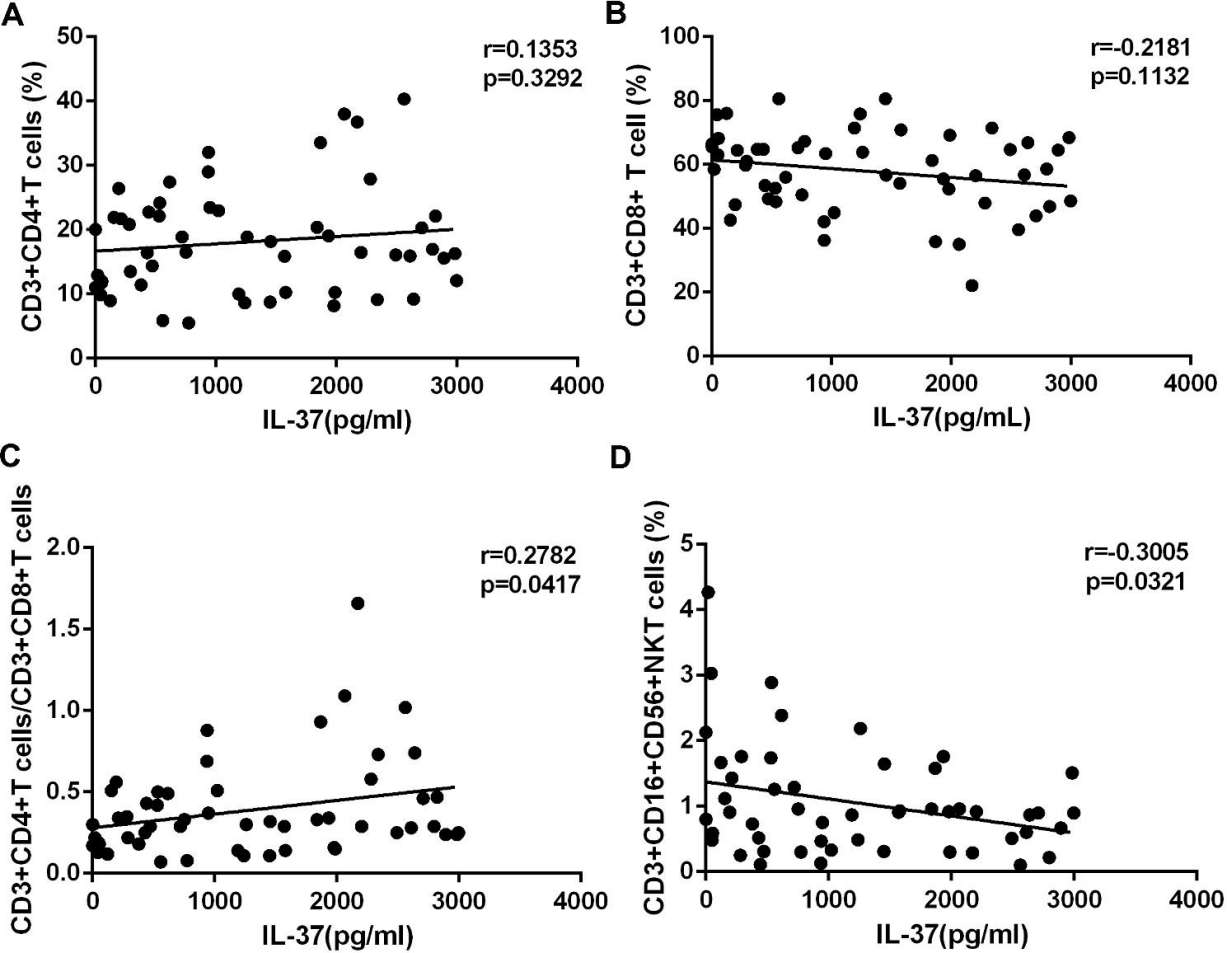
IL-37 expression was positively correlated with the ratio of CD3^+^ CD4^+^ to CD3^+^ CD8^+^ T cells. The correlation between the percentages of CD3^+^CD4^+^T (**A**), CD3^+^CD8^+^T (**B**), CD3^+^CD16^+^CD56^+^NKT (**D**), the ratio of CD3^+^CD4^+^ to CD3^+^CD8^+^T (**C**) and IL-37 expression

The level of immunological function is indicated by the ratio of CD3^+^CD4^+^/CD3^+^CD8^+^T cells. IL-37 expression did not correlate with the frequency of CD3^+^CD4^+^T cells or CD3^+^CD8^+^T cells (Fig. 5A-B), however a significant correlation was found between IL-37 expression and the proportion of CD3^+^CD4^+^ to CD3^+^CD8^+^ T cells (Fig. 5C). Natural killer T (NKT) cells that are CD3^+^CD16^+^CD56^+^ have the same capabilities as CD8 + cytotoxic T cells to destroy the targeted cells, in addition to releasing Th1 and Th2 cytokines. We observed an inverse relationship between IL-37 expression and the proportion of CD3^+^CD16^+^CD56^+^NKT cells in the samples (Fig. 5D).

### IL-37 expression was substantially related to markers of liver injury in patients with IM

**Fig. 6:**
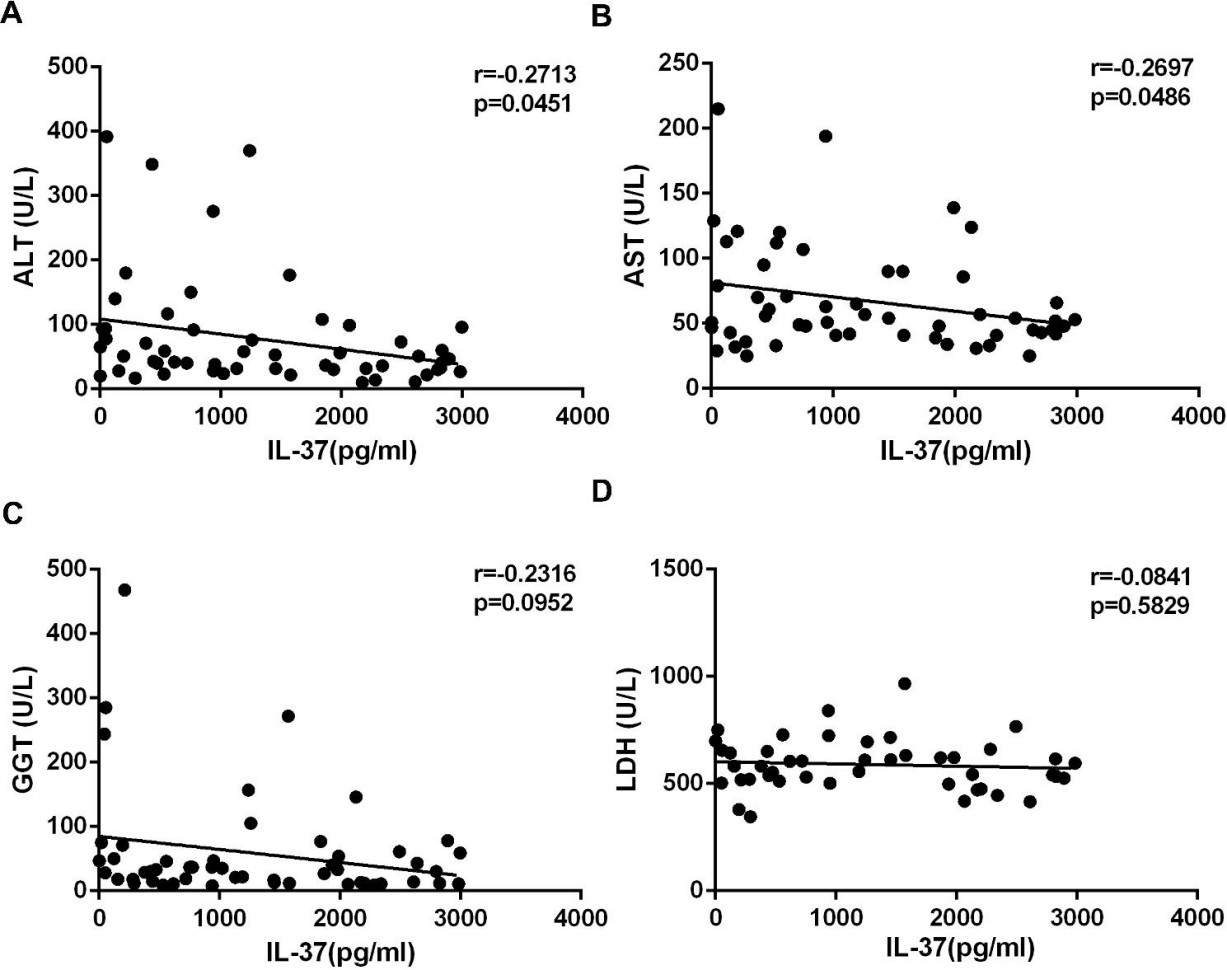
There was a significant correlation between ALT, AST levels and IL-37 expression in patients with IM. The correlation between ALT (**A**), AST levels (**B**), GGT (**C**), LDH levels (**D**) and IL-37 expression

Hepatocytes may be affected by mediators produced in the liver in response to the attack on EBV-infected B lymphocytes by cytotoxic T lymphocytes that have infiltrated the liver parenchyma [15]. The results showed that increased serum alanine aminotransferase (ALT) and aspartate aminotransferase (AST) levels had a significantly inverse relationship with the expression of IL-37 (Fig. 6A-B). Abnormal levels of γ-glutamyl transpeptidase (GGT) and lactate dehydrogenase (LDH) were also observed in some patients, but there was no correlation between them and IL-37 expression (Fig. 6C-D).

## Discussion

IM, one of the most prevalent infections affecting children, is mainly triggered by primary EBV infection. The main target of EBV are B lymphocytes, which can express various antigens on their cell surface. These antigens are subsequently presented to specific T lymphocytes, activating CD8 + T lymphocytes, and causing cytotoxicity of infected B cells [16, 17]. CD3 is the surface marker that identifies all T cells, whereas CD4 is the marker that identifies helper T cells (Th) and CD8 is the marker that identifies effector T cells (cytotoxic T cells, CTL). CD4^+^T cells can secrete various cytokines, which can upregulate the immune system by activating B cells and effector T cells, respectively. Immune response is determined not only by the overall number of lymphocytes but also by the ratio of CD3^+^CD4^+^T cells to CD3^+^CD8^+^T cells (CD3^+^CD4^+^/CD3^+^CD8^+^). This study revealed that the frequency of CD3^+^T and CD3^+^CD8^+^T cells was significantly higher, while the frequency of CD3^+^CD4^+^T cells was significantly lower than in the control group, resulting in a decreased ratio of CD3^+^CD4^+^T cells to CD3^+^CD8^+^T cells, which suggested that immune disorders existed in children with IM.

Importantly, we found that IL-37 expression was significantly higher in patients with IM than in healthy controls. In addition, the levels of IL-37 were found to have a significant correlation with CD3 + T cells and the ratio of CD3^+^CD4^+^ to CD3^+^CD8^+^, which suggests that IL-37 may assist in restoring the patient’s immunity. Meanwhile, a high expression of IL-37 may offer some degree of protection against the liver inflammation experienced by patients. Our results showed that the percentage of CD3 + CD8 + T cells was increased in IM, which is consistent with the results of Balfour Jr and Hilary Williams that the number of CD8 + T cells was increased and positively correlated with severity of illness [18, 19].

The strong immune response of CD4^+^ and CD8^+^ cells to EBV-infected B cells results in worsening of IM and causing multiple organ damage. Extremely large numbers of these cells are present and widely dispersed throughout the tissues and bloodstream. The massive induction and release of cytokines such lymphotoxin, tumor necrosis factor-α (TNF-α), interleukin (IL)-1β and IL-6 [20] may result in the majority of IM symptoms and indications. Negative feedback control, which is mediated by regulatory T cells (Treg) and immunosuppressive medications, is a crucial component of the immune response. Previous studies have suggested that one of the causes of immunological imbalance in patients with IM may be inadequate immunosuppressive function due to a significant decrease in the number of CD4^+^CD25^+^Treg cells during the acute phase of IM [21, 22].

IL-37 is a recently identified cytokine of the IL-1 family, with major immunosuppressive effects and a novel dual anti-inflammatory mechanism [23]. It can enter the nucleus and form a functional complex with Smad3 to regulate gene transcription, and also transmit anti-inflammatory signals by binding to cell surface receptors (IL-18Ra or IL-1R8), resulting in a significant decrease of many cytokines (IL-1β, IL-6, TNF-α, IL-18, etc.) [6, 24]. IL-37 has a protective effect in various inflammatory and autoimmune illnesses by inhibiting excessive inflammation. Recently, Qi et al. reported that IL-37 protected mice from lung injury and enhanced survival in an H1N1 infection model by lowering the release of inflammatory cytokines [25]. This was accomplished by inhibiting the production of IL-1. We found that patients with IM who had higher IL-37 expression had a lower frequency of CD3 + T cells but a higher ratio of CD3 + CD4 + T cells to CD3 + CD8 + T cells. This finding suggests that IL-37 may downregulate the massive immune response in acute IM. Natural killer T (NKT) cells are a subtype of T cells that can produce numerous cytokines and exert both immunostimulatory and cytotoxic effects [26]. NKT cells have both T cell receptor and NK cell receptor on their surface. NKT cell activation is commonly followed by the activation of T cells, B cells, and NK cells [26]. Our findings indicated that IL-37 expression was inversely related to the frequency of CD3 + T cells and CD3 + CD16 + CD56 + NKT cells, suggesting that IL-37 may diminish the excessive inflammation in patients with IM that is created by the excessive proliferation of T and NKT cells.

Acute infections such as IM typically involve the liver, and are distinguished by a transient elevation followed by a decline in the activity of liver enzymes [27]. At the time of a child’s initial EBV infection, severe hepatitis may develop if the immunity is compromised. Although EBV cannot infect hepatocytes directly, these cells may be harmed indirectly by mediators created in the liver due to the attack on EBV-infected B lymphocytes by cytotoxic T lymphocytes that have entered the hepatic parenchyma [15]. Since ALT and AST mainly exist in hepatocytes and are released into the blood when hepatocytes are injured, they are sensitive indicators of acute liver injury. We found that patients with increased IL-37 expression had less liver inflammation, as measured by lower ALT and AST levels. GGT, another important indicator of liver injury, was increased in some patients. IL-37 expression was correlated with GGT levels, but without statistical significance. No significant changes were detected in other parameters of liver function except LDH, perhaps because the patients were in the early stage of liver injury.

There is currently no treatment for IM. While valacyclovir can reduce EBV copy number, it has no effect on the clinical course of the disease [5, 28]. Our data showed no association between IL-37 expression and EBV copy number in the peripheral blood, confirming this clinical observation. IL-37 may provide a potential therapeutic target for IM immunotherapy.

## Conclusions

IL-37 production correlates with the diminution of the immune response against EBV in acute IM, along with decreased liver inflammation. These findings expand our understanding of the immune response to primary EBV infection and offer novel therapeutic strategies for preventing EBV-associated complications.

## Data Availability

Not applicable.

## Abbreviations

IM: infectious mononucleosis
EBV: Epstein-Barr virus
IL-37: interleukin-37
LPS: lipopolysaccharide
ELISA: enzyme-linked immunosorbent assay
ALT: alanine aminotransferase
AST: aspartate aminotransferase
LDH: lactate dehydrogenase
GGT: γ-glutamyl transpeptidase
Th: helper T cells
CTL: cytotoxic T cells
NKT: natural killer T
TNF-α: tumor necrosis factor-α
